# Impact of an Online Gastrointestinal Symptom History Taker on Physician Documentation and Charting Time: Pragmatic Controlled Trial

**DOI:** 10.2196/23599

**Published:** 2021-05-04

**Authors:** Natalie J Hall, Sameer K Berry, Jack Aguilar, Elizabeth Brier, Parth Shah, Derek Cheng, Jeremy Herman, Theodore Stein, Brennan M R Spiegel, Christopher V Almario

**Affiliations:** 1 Department of Medicine Cedars-Sinai Medical Center Los Angeles, CA United States; 2 Karsh Division of Gastroenterology and Hepatology Cedars-Sinai Medical Center Los Angeles, CA United States; 3 Division of Health Services Research Cedars-Sinai Medical Center Los Angeles, CA United States; 4 Division of Informatics Cedars-Sinai Medical Center Los Angeles, CA United States; 5 Cedars-Sinai Center for Outcomes Research and Education (CS-CORE) Los Angeles, CA United States

**Keywords:** patient-provider portal, computer-generated patient history, patient-reported outcome, gastrointestinal, EHR

## Abstract

**Background:**

A potential benefit of electronic health records (EHRs) is that they could potentially save clinician time and improve documentation by auto-generating the history of present illness (HPI) in partnership with patients prior to the clinic visit. We developed an online patient portal called AEGIS (Automated Evaluation of Gastrointestinal [GI] Symptoms) that systematically collects patient GI symptom information and then transforms the data into a narrative HPI that is available for physicians to review in the EHR prior to seeing the patient.

**Objective:**

This study aimed to compare whether use of an online GI symptom history taker called AEGIS improves physician-centric outcomes vs usual care.

**Methods:**

We conducted a pragmatic controlled trial among adults aged ≥18 years scheduled for a new patient visit at 4 GI clinics at an academic medical center. Patients who completed AEGIS were matched with controls in the intervention period who did not complete AEGIS as well as controls who underwent usual care in the pre-intervention period. Of note, the pre-intervention control group was formed as it was not subject to contamination bias, unlike for post-intervention controls. We then compared the following outcomes among groups: (1) documentation of alarm symptoms, (2) documentation of family history of GI malignancy, (3) number of follow-up visits in a 6-month period, (4) number of tests ordered in a 6-month period, and (5) charting time (difference between appointment time and time the encounter was closed). Multivariable regression models were used to adjust for potential confounding.

**Results:**

Of the 774 patients who were invited to complete AEGIS, 116 (15.0%) finished it prior to their visit. The 116 AEGIS patients were then matched with 343 and 102 controls in the pre- and post-intervention periods, respectively. There were no statistically significant differences among the groups for documentation of alarm symptoms and GI cancer family history, number of follow-up visits and ordered tests, or charting time (all *P*>.05).

**Conclusions:**

Use of a validated online HPI-generation portal did not improve physician documentation or reduce workload. Given universal adoption of EHRs, further research examining how to optimally leverage patient portals for improving outcomes are needed.

## Introduction

To facilitate communication between patients and physicians in electronic health record (EHR)–integrated environments, we developed an online patient portal (MyGiHealth) that uses a computer algorithm called Automated Evaluation of Gastrointestinal (GI) Symptoms (AEGIS) to systematically collect patients’ symptom information before the clinic visit. Once collected, the data are transformed into a full narrative history of present illness (HPI) that clinicians can review prior to meeting the patient. While our prior studies noted that AEGIS creates higher quality HPIs and collects more alarm features vs physicians [1,2], we found that it did not improve patient satisfaction or shared decision making when compared to usual care in a controlled trial [3]. Therefore, the objective of this study was to investigate whether AEGIS improved physician-centric outcomes vs usual care.

## Methods

We performed a pragmatic controlled study among adults aged ≥18 years scheduled for a new patient visit at an academic GI teaching practice and 3 community-based GI clinics at Cedars-Sinai Medical Center. This study was approved by the Cedars-Sinai Institutional Review Board, Los Angeles, CA (Pro45243).

**Figure 1:**
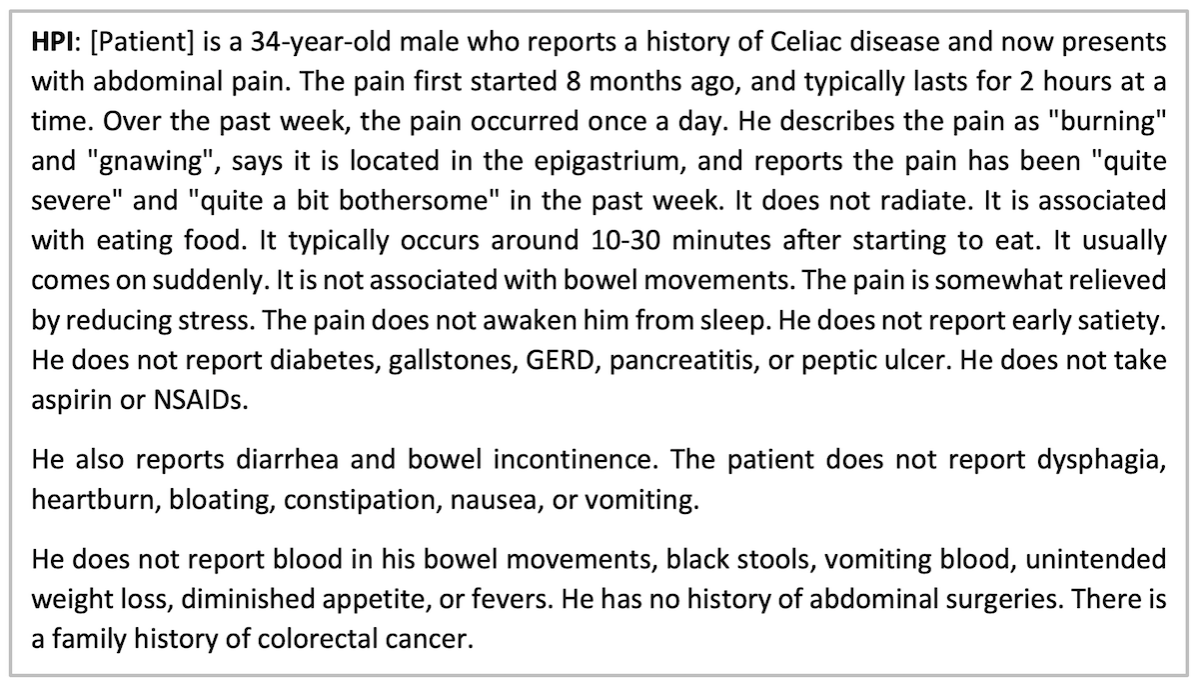
Sample Automated Evaluation of Gastrointestinal Symptoms (AEGIS) history of present illness (HPI) [3], which was composed entirely by the AEGIS software and based on the patient’s responses to questions about their gastrointestinal symptoms; then, the HPI is uploaded into the electronic health record where the physician can review it prior to seeing the patient as well as copy the HPI into their consult note and modify it as needed based on the subsequent clinical encounter. GERD: gastroesophageal reflux disease; NSAID: nonsteroidal anti-inflammatory drug.

During the intervention period (April 17, 2017-February 7, 2018), patients were invited via email to complete AEGIS via the MyGiHealth app 1 week prior to their visit. We describe AEGIS elsewhere [1-4], but in brief, the algorithm systematically assesses patients’ GI symptoms and then transforms the data into a full narrative HPI as shown in Figure 1. For patients who completed AEGIS, their physicians were notified 1 day before the visit that their HPIs were uploaded to the notes section of our EHR (Epic, Verona, WI) for review (see Multimedia Appendix 1 for the email notification that was sent to physicians by research study staff). To identify individuals for the 2 control groups who were comparable to those in the intervention arm, each patient who completed AEGIS was matched (age ±3 years, sex, race/ethnicity, clinic) with up to 4 patients in the pre-intervention period (October 6, 2015-April 6, 2017) and 1 patient in the intervention period who did not complete AEGIS. Of note, the pre-intervention control group was formed as it was not subject to contamination bias, unlike for post-intervention controls (ie, after physicians reviewed AEGIS reports for those in the intervention arm, they might have been more apt to take and document more thorough HPIs for their control patients). Moreover, age, sex, race/ethnicity, and clinic were selected as matching variables as they were readily available for automated extraction from the EHR and we hypothesized at the outset that they may have correlated with our outcomes.

Once recruitment ceased, 5 resident physicians reviewed patient charts and collected outcomes data using a REDCap data abstraction sheet [5]: (1) documentation of alarm symptom(s) in initial note (hematochezia, melena, hematemesis, unintentional weight loss, fevers); (2) documentation of family history of GI malignancy in initial note (colorectal, esophagus, gallbladder, liver, stomach, pancreas, or throat cancer); (3) charting time (time initial encounter closed minus the appointment time); (4) number of follow-up visits in a 6-month period; (5) number of lab, endoscopy, and imaging tests ordered in a 6-month period.

Statistical analyses were performed using Stata 13.1 (StataCorp LP, College Station, TX). A two-tailed *P*<.05 was considered significant in all analyses. For bivariate analyses, we used analysis of variance and chi-squared tests to compare continuous and categorical data, respectively. We also conducted multivariable regression analyses on our outcomes and included group assignment, patient age, sex, race/ethnicity, and clinic site as covariates when appropriate. Specifically, logistic regression analyses were performed on the AEGIS completion and documentation of alarm symptoms and GI cancer family history outcomes. We used linear regression analysis to compare charting time between the intervention and control groups. Lastly, numbers of follow-up visits and tests ordered within 6 months of the initial visit were compared using zero-inflated negative binomial and negative binomial regression models, respectively.

## Results

Of the 774 patients invited to complete AEGIS (Multimedia Appendix 2 shows demographics), 116 (15.0%) completed it before their clinic visit. Table 1 shows results from the regression analysis on completion of the app; older individuals and Asians were less likely to complete AEGIS. No significant associations were seen between app completion and the remaining racial/ethnic groups, sex, and clinic. Among those who completed AEGIS, the consultants’ initial notes for 47 (40.5%) of the 116 patients contained at least a portion of the computer-generated report.

**Table 1:** Predictors of completing the Automated Evaluation of Gastrointestinal Symptoms (AEGIS) prior to the clinic visit (N=774).

Variable			Completed AEGIS (n=116)		OR^a^ (95% CI)^b^
Age (years), mean (SD)			49.9 (16.1)		0.985 (0.972-0.998)
**Sex, n (%)**					
	Male	45 (14.9)		reference	
	Female	71 (15.1)		0.99 (0.66-1.51)	
**Race/ethnicity, n (%)**					
	Non-Hispanic white	83 (17.9)		Reference	
	Non-Hispanic black	11 (11.0)		0.57 (0.29-1.13)	
	Latino	10 (13.7)		0.66 (0.32-1.36)	
	Non-Hispanic Asian	5 (7.3)		0.33 (0.13-0.85)	
	Other/unknown	7 (10.5)		0.48 (0.21-1.11)	
**Clinic, n (%)**					
	Resident/fellow GI^c^ clinic	19 (17.4)		Reference	
	Physician A	37 (18.2)		1.08 (0.58-2.02)	
	Physician B	29 (12.7)		0.68 (0.36-1.30)	
	Physician C	31 (13.3)		0.74 (0.38-1.42)	

^a^OR: odds ratio.

^b^The logistic regression model adjusted for all covariates in the table.

^c^GI: gastrointestinal.

Patients who completed AEGIS (n=116) were matched with 343 patients from the pre-intervention period and 102 from the intervention period who did not complete AEGIS. Table 2 lists their demographics; the groups were largely similar in age, sex, race/ethnicity, clinic, and reason for consult.

**Table 2:** Demographics of those in the matched cohort analysis (N=561).

Variable			Control group: pre-AEGIS^a^ period (n=343)		Control group: did not complete AEGIS (n=102)		Intervention group: completed AEGIS (n=116)		*P* ^b^
Age (years), mean (SD)			51.4 (16.1)		53.7 (16.2)		49.9 (16.1)		.21
**Sex, n (%)**									.51
	Male	136 (39.7)		34 (33.3)		45 (38.8)			
	Female	207 (60.4)		68 (66.7)		71 (61.2)			
**Race/ethnicity, n (%)**									.25
	Non-Hispanic white	264 (77.0)		86 (84.3)		83 (71.6)			
	Non-Hispanic black	35 (10.2)		7 (6.9)		11 (9.5)			
	Latino	23 (6.7)		2 (2.0)		10 (8.6)			
	Non-Hispanic Asian	10 (2.9)		5 (4.9)		5 (4.3)			
	Other/unknown	11 (3.2)		2 (2.0)		7 (6.0)			
**Clinic, n (%)**									.67
	Resident/fellow GI^c^ clinic	43 (12.5)		9 (8.8)		19 (16.4)			
	Physician A	112 (32.7)		30 (29.4)		37 (31.9)			
	Physician B	90 (26.2)		28 (27.5)		29 (25.0)			
	Physician C	98 (28.6)		35 (34.3)		31 (26.7)			
**Reason for consult, n (%)**									
	Abdominal pain	77 (22.5)		27 (26.5)		28 (24.1)		.69	
	Anemia evaluation	2 (0.6)		1 (1.0)		0 (0)		.60	
	Bloating	30 (8.8)		17 (16.7)		21 (18.1)		.008	
	Blood in stool	18 (5.3)		2 (2.0)		5 (4.3)		.37	
	Bowel incontinence	2 (0.6)		1 (1.0)		1 (0.9)		.90	
	Colorectal cancer screening	101 (29.5)		25 (24.5)		34 (29.3)		.61	
	Constipation	48 (14.0)		13 (12.8)		19 (16.4)		.73	
	Diarrhea	42 (12.2)		10 (9.8)		17 (14.7)		.55	
	Dysphagia	15 (4.4)		1 (1.0)		4 (3.5)		.27	
	Gastroesophageal reflux disease	46 (13.4)		15 (14.7)		33 (28.5)		.001	
	Inflammatory bowel disease	19 (5.5)		4 (3.9)		6 (5.2)		.81	
	Liver disease	2 (0.6)		1 (1.0)		1 (0.9)		.90	
	Nausea/vomiting	25 (7.3)		9 (8.8)		8 (6.9)		.84	
	Rectal pain	2 (0.6)		0 (0)		2 (1.7)		.29	
	Other	47 (13.7)		19 (18.6)		16 (13.8)		.45	

^a^AEGIS: Automated Evaluation of Gastrointestinal Symptoms.

^b^*P* value from analysis of variance test (continuous data) or chi-squared test (categorical data).

^c^GI: gastrointestinal.

In Table 3, we present the physician-centric outcomes stratified by group. No differences were seen for documentation of alarm symptoms and GI cancer family history, EHR charting time, or numbers of follow-up visits and ordered tests.

**Table 3:** Physician-related outcomes according to study group (N=561).

Variable	Control group: pre-AEGIS^a^ period (n=343; reference)	Control group: did not complete AEGIS (n=102)	Adjusted *P*	Intervention group: completed AEGIS (n=116)	Adjusted *P*
Documentation of an alarm symptom in initial note^b^, n (%)	61 (17.8)	22 (21.6)	.18	18 (15.5)	.39
Documentation of GI^c^ cancer family history in initial note^b^, n (%)	64 (18.7)	20 (19.6)	.86	27 (23.3)	.28
Charting time, which is the time until initial EHR^d^ chart encounter was closed^e^ (hours), median (IQR)	3.1 (1.4-9.2)	3.3 (1.0-12.7)	.34	3.7 (1.1-10.0)	.58
Number of follow-up visits within the 6-month period^f^, median (IQR)	0 (0-1)	0 (0-0)	.22	0 (0-1)	.11
Number of tests ordered within the 6-month period^g^, median (IQR)	1 (1-3)	1 (0-2)	.21	1 (0-3)	.85

^a^AEGIS: Automated Evaluation of Gastrointestinal Symptoms.

^b^Logistic regression model adjusted for group assignment, patient age, sex, race/ethnicity, and clinic.

^c^GI: gastrointestinal.

^d^EHR: electronic health record.

^e^Linear regression model adjusted for group assignment, patient age, sex, race/ethnicity, and clinic. Patients seen in the resident or fellow GI clinic (n=71) were excluded from this analysis as trainees first needed to complete their note before attendings could review or edit the note and close the encounter. Patients of Physicians A-C who were seen earlier or later than their originally scheduled appointment time (n=92) were also excluded from this analysis.

^f^Zero-inflated negative binomial regression model adjusted for group assignment, patient age, sex, race/ethnicity, and clinic.

^g^Negative binomial regression model adjusted for group assignment, patient age, sex, race/ethnicity, and clinic.

## Discussion

We discovered that uptake of AEGIS was low, as only 15% of patients accessed the online portal. Surprisingly, this rate was lower than that seen in our prior AEGIS trial (37%) focused on patient-centric outcomes [3]. This was even despite our use of email invitations (the original study used mailings), which we initially hypothesized would increase uptake as the email included a direct AEGIS hyperlink. Of note, research staff emailed invitations directly to patients; it is possible that sending invitations through the EHR patient portal may have enhanced uptake. Prior literature illustrates that patients are accepting of EHR portals [6-8] and they may be more willing to complete interventions sent through official health system platforms. Further research examining optimal methods for deploying digital interventions in EHR-integrated environments are needed.

While AEGIS was built to enhance patient-physician communication by systematically collecting salient components of the history, one-time use of the app did not increase documentation of alarm symptoms or family history of GI malignancy in the initial note. This suggests that physicians in our study may adequately screen for and document relevant red flags and family history*.* Alternatively, given our pragmatic design, clinicians may not have reviewed the AEGIS report or incorporated it into their note for some patients. We also noted that the app and its computer-generated HPIs did not impact health care utilization or charting time. In short, we did not find that leveraging an online HPI-generation portal measurably improved physician-centric outcomes in this study.

Of note, we previously found that AEGIS collected more alarm features when compared to physicians [2]. The discordant results likely relate to the different study designs; in the prior observational study, AEGIS was completed by patients after their clinic visit, rather than before the visit as in our current study. It is possible that first consulting with the physician subsequently prompted patients, after further introspection, to report more alarm features through AEGIS than were discussed and documented by the physician in clinic. We also previously found that AEGIS creates higher quality HPIs versus those written by doctors [1]. However, based on our findings here and in a prior multicenter controlled trial focused on patient-centered outcomes [3], simply making the comprehensive AEGIS HPIs available for review in the EHR is insufficient for improving care. Further research is needed to determine how best to optimally implement and use these computer-generated data in clinical workflows in order to enhance outcomes.

A limitation of our study was that we could not fully assess whether and how closely physicians reviewed the AEGIS reports in the EHR. While 40.5% of physicians’ notes for the intervention patients contained a portion of the AEGIS report, we do not know how rigorously they reviewed the report after copying it into their notes. On the other hand, for the remaining patients, it is possible that clinicians thoroughly read the AEGIS report in the EHR but chose to not copy and paste it into their official consultant notes. Development of novel, effective methods for alerting and assessing how clinicians use newly uploaded, app-generated data in the EHR and that maximize its use at the point of care are urgently needed. Another limitation was that AEGIS was administered as a one-time intervention; longitudinal use of the app for tracking symptom severity could have impacted outcomes such as numbers of follow-up visits and ordered tests. Notably, longitudinal symptom monitoring via a portal decreased emergency room visits and improved survival among patients with metastatic cancer [9,10].

In summary, we found that uptake of AEGIS was low, as less than 1 in 6 patients completed it before their visit. Moreover, one-time use of a carefully developed and validated patient-provider portal did not improve documentation of key elements of the note nor reduce clinician work burden. This is disappointing as it is well known that the EHR has greatly increased physician charting time [11,12]; our goal has been to identify ways to reduce physician burden through clinically meaningful, EHR-enabled automation. Yet, even in taking care to maximize the benefits of the EHR to support physician-centric outcomes, we were unable to demonstrate a benefit. Given the near universal adoption of EHRs [13-15], further research examining how best to develop and implement digital interventions in EHR-integrated environments for improving both patient and physician outcomes is critical.

## Appendix

Multimedia Appendix 1 Supplementary Figure 1. Sample email sent to physicians the day before their clinic notifying them of the patients that completed AEGIS.

Multimedia Appendix 2 Supplementary Table 1. Demographics of the cohort invited to complete AEGIS (N=774).

## Abbreviations

AEGIS: Automated Evaluation of Gastrointestinal Symptoms
EHR: electronic health record
GI: gastrointestinal
HPI: history of present illness
OR: odds ratio
