# Supplementary figures and images for: Impact of an Online Gastrointestinal Symptom History Taker on Physician Documentation and Charting Time: Pragmatic Controlled Trial
Source: JMIR Form Res. 2021 May 4;5(5):e23599. doi: 10.2196/23599 (PMC8132977; doi:10.2196/23599)

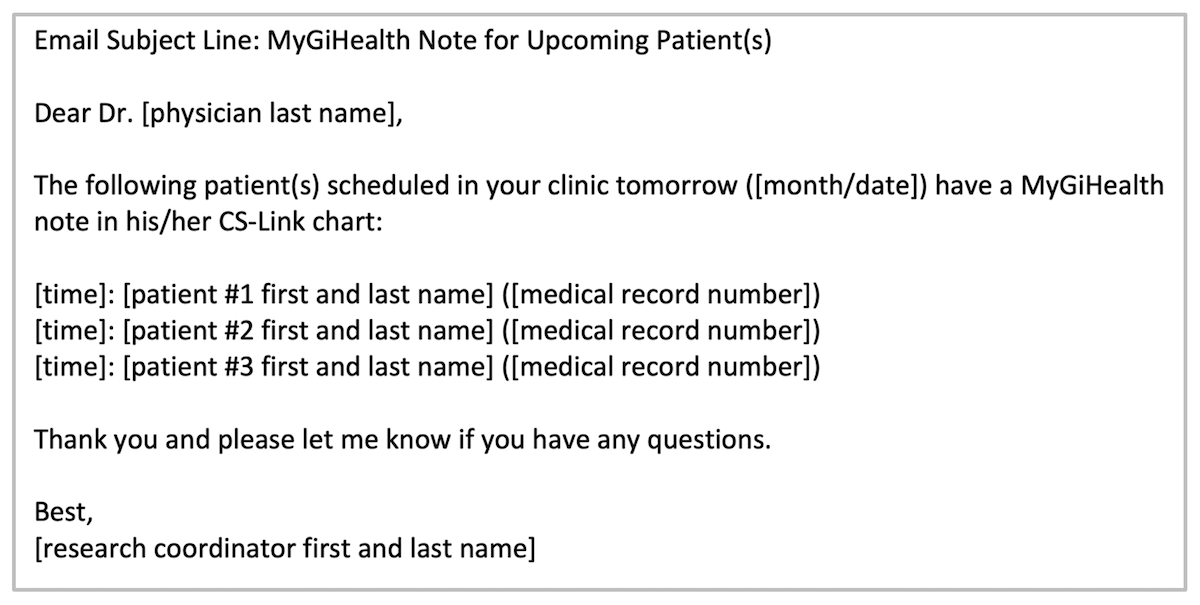

Supplement: Multimedia Appendix 1 [file formative_v5i5e23599_app1.png]
