# Supplementary material for: Impact of an Online Gastrointestinal Symptom History Taker on Physician Documentation and Charting Time: Pragmatic Controlled Trial
Source: JMIR Form Res. 2021 May 4;5(5):e23599. doi: 10.2196/23599 (PMC8132977; doi:10.2196/23599)
Supplement: Multimedia Appendix 2 [file formative_v5i5e23599_app2.docx]

**SUPPLEMENTARY TABLE 1.** Demographics of the cohort invited to complete AEGIS (N=774).

| **Variable** | **Mean (standard deviation) or n (%)** |
| --- | --- |
| Age (years) | 53.3 (15.6) |
| Sex: |  |
| Male | 303 (39.2%) |
| Female | 471 (60.9%) |
| Race/ethnicity: |  |
| Non-Hispanic white | 465 (60.1%) |
| Non-Hispanic black | 100 (12.9%) |
| Latino | 73 (9.4%) |
| Non-Hispanic Asian | 69 (8.9%) |
| Other/unknown | 67 (8.7%) |
| Clinic: |  |
| Resident/fellow GI clinic | 109 (14.1%) |
| Physician A | 203 (26.2%) |
| Physician B | 229 (29.6%) |
| Physician C | 233 (30.1%) |
| AEGIS, Automated Evaluation of Gastrointestinal Symptoms; GI, gastrointestinal. | |
